# Semi-Annual Meeting of the Erie Co. Medical Society

**Published:** 1847-08

**Authors:** 


*Semi-Annual Meeting of the Erie Co. Medical Society.*—We omitted in our last No. to notice the semi-annual meeting of the Medical Society of this County, which was holden in June last. Of the proceedings of the meeting we recollect nothing of particular interest, except the appointment of a committee to correspond with members of the profession in different portions of the State, with reference to an united effort to procure the passage of laws sanctioning and providing for dissections. This action was taken in conformity with a resolution adopted by the late Convention. We propose to offer some remarks on this highly important subject in our next number.

The address was by Dr. Baily of this city; subject, Medical Education. We were unable to be present at the delivery of the address, but we are informed that it was creditable to the writer.

